# Identification of arthritis-related gene clusters by microarray analysis of two independent mouse models for rheumatoid arthritis

**DOI:** 10.1186/ar1985

**Published:** 2006-06-28

**Authors:** Noriyuki Fujikado, Shinobu Saijo, Yoichiro Iwakura

**Affiliations:** 1 Center for Experimental Medicine, Institute of Medical Science, University of Tokyo, 4-6-1 Shirokanedai, Minato-ku, Tokyo 108-8639, Japan

## Abstract

Rheumatoid arthritis (RA) is an autoimmune disease affecting approximately 1% of the population worldwide. Previously, we showed that human T-cell leukemia virus type I-transgenic mice and interleukin-1 receptor antagonist-knockout mice develop autoimmunity and joint-specific inflammation that resembles human RA. To identify genes involved in the pathogenesis of arthritis, we analyzed the gene expression profiles of these animal models by using high-density oligonucleotide arrays. We found 1,467 genes that were differentially expressed from the normal control mice by greater than threefold in one of these animal models. The gene expression profiles of the two models correlated well. We extracted 554 genes whose expression significantly changed in both models, assuming that pathogenically important genes at the effector phase would change in both models. Then, each of these commonly changed genes was mapped into the whole genome in a scale of the 1-megabase pairs. We found that the transcriptome map of these genes did not distribute evenly on the chromosome but formed clusters. These identified gene clusters include the major histocompatibility complex class I and class II genes, complement genes, and chemokine genes, which are well known to be involved in the pathogenesis of RA at the effector phase. The activation of these gene clusters suggests that antigen presentation and lymphocyte chemotaxisis are important for the development of arthritis. Moreover, by searching for such clusters, we could detect genes with marginal expression changes. These gene clusters include schlafen and membrane-spanning four-domains subfamily A genes whose function in arthritis has not yet been determined. Thus, by combining two etiologically different RA models, we succeeded in efficiently extracting genes functioning in the development of arthritis at the effector phase. Furthermore, we demonstrated that identification of gene clusters by transcriptome mapping is a useful way to find potentially pathogenic genes among genes whose expression change is only marginal.

## Introduction

Rheumatoid arthritis (RA) is a systemic, chronic inflammatory disease primarily affecting the joints. The synovial inflammation leads to cartilage destruction, bone erosion, joint deformity, and loss of joint function [1]. This disease is autoimmune in nature and characterized by the infiltration of T cells, B cells, macrophages, and neutrophils into the synovial lining and fluid of the periarticular spaces [2]. The infiltrating cells express adhesion molecules and produce a variety of inflammatory cytokines and chemokines to contribute to the complex pathogenesis of RA. The etiopathogenesis of this disease has not yet been completely elucidated.

Using gene-manipulating techniques, we have established two mouse models for RA: human T-cell leukemia virus type I (HTLV-I)-transgenic (Tg) mice and interleukin-1 receptor antagonist (IL-1Ra)-knockout (KO) mice [3,4]. HTLV-I is the causative agent of adult T-cell leukemia. The virus encodes a transcriptional transactivator, Tax, within the *pX *region that activates multiple cellular genes, including those for cytokines, cytokine receptors, and immediate early transcriptional factors, via activation of enhancers such as cAMP-responsive enhancer, nuclear factor kappa B-dependent enhancers, or serum-responsive elements [5,6]. Tg mice carrying the *tax *gene spontaneously develop autoimmune arthritis, likely due to overexpression of proinflammatory cytokines and increased T-cell resistance to Fas-induced apoptosis [2,3,7]. IL-1Ra is a negative regulator of IL-1 which competes for the binding of IL-1α and IL-1β to its cognate receptors. Because the three isoforms of IL-1Ra protein, which possess inhibitory activity against IL-1, are synthesized by alternative splicing of a single gene, we produced mice deficient in all three isoforms of IL-1Ra. These IL-1Ra-KO mice also spontaneously develop autoimmune arthritis, due to excess T-cell activation [2,4,8].

Although the etiology of the arthritis differs between these mice, the histopathologies of the lesions are very similar. These lesions exhibit marked synovial and periarticular inflammation, with articular erosion caused by the invasion of granulation tissues, which closely resembles RA in humans. Osteoclast activation is obvious at the pannus, and the infiltration of inflammatory cells, including neutrophils, lymphocytes, and macrophages, can be detected in synovial tissues. Both of these mouse models develop autoimmunity with elevated antibody titers against immunoglobulin (Ig) G and type II collagen. Given that the histopathology observed in these models closely resembles that seen in RA in humans, pathogenic mechanisms similar to those operating in these models are likely functioning in human RA. Actually, an etiological correlation was suggested between HTLV-I and RA in Japan [9,10]. In addition, an association was suggested between IL-1Ra polymorphism and RA [11,12]. We took advantage of these mouse models of RA to analyze comprehensively the gene expression patterns functioning in this condition, using high-density oligonucleotide arrays.

In this analysis, we focused on genes that exhibited similar changes in both of the disease models. This approach should efficiently identify the genes involved in the pathogenesis of arthritis irrespective of the etiology, and these genes should include those that function in the effector phase of inflammation or in the bone erosion process. To determine the genomic distribution of the arthritis-related genes, we assigned these genes into the whole genome. The members of the same gene family often form clusters on the chromosomes [13,14]. Furthermore, because relatively wide genomic regions form open complex structures upon activation [15,16], we expected that genes in the same cluster might be functionally related. Using this analysis, we identified several arthritis-related gene clusters in the specific genomic regions, and some of the genes were successfully detected as a cluster whose expression changes are only marginal.

## Materials and methods

### Mice

Two mouse models were used for gene expression profiling studies. HTLV-I-Tg mice, originally produced by injection of the LTR-*env-pX*-LTR region of the HTLV-I genome into a (C3H/Hen x C57BL/6J) F_1 _embryo [3], were backcrossed to BALB/cA mice (CLEA Japan, Inc., Tokyo, Japan) for 20 generations. These mice start to develop arthritis spontaneously at 4 weeks of age, and 60% and 80% of the mice are affected at 3 months and 6 months of age, respectively. In this study, severely arthritic (score 3) HTLV-I-Tg mice (TS) (females, 6 to 9 weeks of age) were used. Wild-type (WT) littermates were used as controls. IL-1Ra-KO mice, generated by homologous recombination as described previously [4], were backcrossed to BALB/cA mice for eight generations. These mice develop arthritis spontaneously at 5 weeks of age. Eighty percent and almost 100% of the mice became arthritic by 8 and 13 weeks of age, respectively. In this study, IL-1Ra-KO mice (male, 13 weeks of age) that suffered from severe arthritis (score 3) (KS) were used; WT littermates were used as controls. The severity of arthritis was graded for each paw on a scale of 0 to 3 for the degree of redness and swelling: grade 0 = normal; grade 1 = light swelling of the joint and/or redness of the footpad; grade 2 = obvious joint swelling; and grade 3 = severe swelling and fixation of the joint. All mice were kept under specific pathogen-free conditions in an environmentally controlled clean room at the Center for Experimental Medicine, Institute of Medical Science, University of Tokyo. Experiments were conducted according to the institutional ethical guidelines for animal experimentation and the safety guidelines for gene manipulation.

### Preparation of total RNA and poly (A)^+ ^RNA from joints

After removal of the skin and muscle, portions of the leg containing the knee, ankle, and finger joints were rapidly frozen in liquid nitrogen. Frozen joints were homogenized using a physcotron (Microtech, Chiba, Japan). Total RNA was extracted from the joint homogenate using the acid guanidium thiocyanate/phenol chloroform extraction method. To avoid fluctuation between individuals, total joint RNAs were pooled from three (TS) or five (KS) arthritic mice and five or six (WT) normal mice for replicates; poly (A)^+ ^RNA was purified on oligo (dT)-cellulose columns. RNA quality was confirmed by spectrophotometry and electrophoresis on formaldehyde gels.

### Microarray analysis

A Murine Genome U74v2 Set (GeneChip^® ^system; Affymetrix, Santa Clara, CA, USA) consisting of three GeneChip probe arrays containing approximately 36,000 oligonucleotide probe sets (6,000 full-length mouse genes and 30,000 expressed sequence tag (EST) clusters from the UniGene database) was used for the analysis. Sample labeling and processing were performed according to the manufacturer's protocol. In brief, double-stranded complementary DNA was synthesized, and biotinylated cRNA was prepared and then hybridized to the GeneChip sets. Fluorescent hybridization signals were developed with phycoerythrin-conjugated streptavidin. Fluorescent signals were collected by laser scan, and the results were analyzed with GENECHIP ANALYSIS software (Affymetrix).

### Northern blot hybridization analysis

Tissues were quickly frozen in liquid nitrogen and stored at -80°C. Frozen tissues were homogenized with a physcotron (Microtech). Total RNA was isolated from tissue homogenates by an acid guanidium thiocyanate-phenol-chloroform extraction method, and poly (A)^+ ^RNA was purified using an oligo (dT)-cellulose column. The poly (A)^+ ^RNA was extracted from the paws of four to five mice. Then, the poly (A)^+ ^RNA was electrophoresed on a 1.3% denatured agarose gel and transferred to a nylon membrane (Gene Screen Plus; NEN Life Science, Boston, MA, USA). Hybridization was performed at 42°C overnight with ^32^P-labeled DNA probes labeled with Megaprime DNA labeling system (GE Healthcare, Little Chalfont, Buckinghamshire, UK) and ^32^P-dCTP (3,000 Ci/mmol; NEN Life Science). The radioactivity was measured using the BAS-2000 system (Fuji Photo Film Co., Tokyo, Japan).

### Statistical analysis and data management

Data were normalized by the average fluorescent intensities for each microarray experiment, and expression values based on the perfect match/mismatch model were calculated for each GeneChip. For the pairwise comparison between normal mice and arthritic mice, signals were filtered using several criteria. The following gene sets were selected: (a) the gene was present in the arthritic mice but absent in the normal mice, (b) the gene was present in the normal mice but absent in the arthritic mice, and (c) the gene was present in both arthritic mice and normal mice. Fold changes for gene expression were calculated, and genes with more than a threefold change in gene expression were selected for further characterization. We assumed that the same group of genes is involved in the pathogenesis of arthritis in both models, and we extracted commonly changed genes in both models. To extract commonly changed genes, we applied the principle of the significance analysis of microarrays (SAM) method [17] for the statistical analysis of the microarray data. SAM assigns a score, *d*(*i*), to each gene on the basis of changes in gene expression relative to the standard deviation (SD) of repeated measurements. The 'relative difference', *d*(*i*), in gene expression is defined as:



where _*A*_(*i*) and _*N*_(*i*) are the average expression levels of a gene (*i*) in subjects A (arthritis) and N (normal), respectively. The 'gene-specific scatter', *s*(*i*), is the SD of the expression measurements. At low expression levels, the variance *d*(*i*) can be high because of the small values of *s*(*i*). To avoid this, SAM defines a small positive constant, *s*_0_, in the denominator of the above equation. For the present data, computation yielded *s*_0 _= 0.11. The *d*(*i*) is calculated for each gene, and significance levels are indicated by the *q *value. The *q *value is the lowest false discovery rate, the probability of being identified by chance [17]. To assess the reliability of the data, the mean values of the fold change and SD were calculated for the genes that were more than two in the selected gene sets. The range of variance was estimated by calculating ratios of SD to fold change. Forty genes exhibited duplicate or triplicate clones. A conservative approach of interval estimation was used to estimate the dispersion of the fold-change values. The estimated ratio was 0.20 (95% confidence interval, 0.15 to 0.25). This result signifies that the fold-change data fluctuated approximately 20% in this study.

### Gene mapping

Using the public genome database [18], SOURCE database [19], and Mouse Genome Search in The Bioinformatics Analytical Toolkit [20], we identified the chromosome and the genomic position at which the genes localized for 538 of the 554 significant genes. Gene cluster scanning was performed in each 1-megabase (Mb) window. Hierarchical clustering was applied using 'Cluster' software, and the results were visualized with 'Treeview' software (M. Eisen at Stanford University, Stanford, CA)[21,22].

## Results

### Gene expression profiles of synovial tissues from RA model mice compared with normal control mice

**Figure 1:**
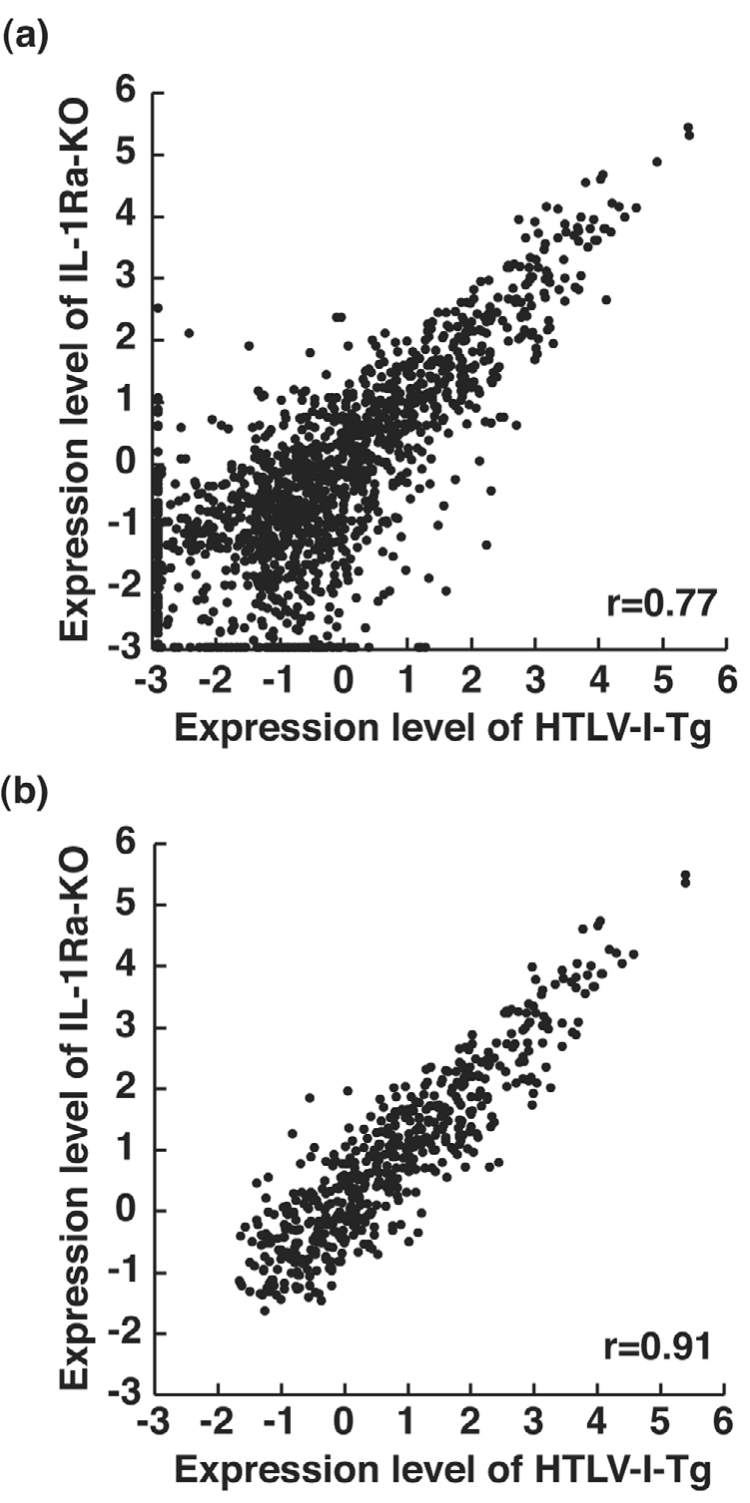
The relationship of gene expression levels between human T-cell leukemia virus type I- transgenic (HTLV-I-Tg) and interleukin-1 receptor antagonist-knockout (IL-1Ra-KO) mice. **(a)** The reciprocal relationship between log-transformed (base 2) normalized gene expression levels for the two models was plotted. The correlation coefficient of gene expression between HTLV-I-Tg and IL-1Ra-KO mice is *r *= 0.77. **(b)** Commonly activated genes were extracted in different models using the SAM (significance analysis of microarrays) method; their relationship is shown. The correlation coefficient is *r *= 0.91.

We isolated mRNA from the joints of two arthritic models (HTLV-I-Tg and IL-1Ra-KO mice) and normal WT mice. Isolated RNAs were labeled and hybridized to microarrays containing the oligonucleotide probes of approximately 36,000 mouse genes and ESTs from the UniGene database. In this microarray system, each gene is represented as 16 distinct pairs of 25-mer oligonucleotide probes. The mismatch oligonucleotide provides an estimate of the background hybridization signal. Fluctuation of the fold-change data of the genes that were spotted more than once on the array was only 20% (see Materials and methods). Therefore, these arrays allow highly reproducible quantification of gene expression levels. We set the threshold at threefold change, which is well above the fluctuation. We performed two independent experiments for control mice, using independently pooled mRNA preparations from five and six mice, respectively, and the reproducibility was confirmed. Fluctuation between two experiments was approximately 11%. For arthritic mice, mRNA was prepared from three HTLV-I-Tg mice and five IL-1Ra-KO mice, respectively. Accordingly, we selected 1,467 genes, for which the transcript levels changed at least threefold in one of either model from that of the corresponding WT normal mice (Additional File 1). When the log-transformed (base 2) normalized intensities of gene expression levels in HTLV-I-Tg mice were plotted against those of IL-1Ra-KO mice, we observed a high degree of correlation (correlation coefficient, *r *= 0.77), indicating that the expression of many genes changed in both models (Figure 1).

We thought that the same group of genes should be involved in the development of arthritis in both models at the effector phase because the pathology of the disease is very similar between two models. We searched for genes for which the expression levels in the joints changed significantly in both models in comparison with those seen in normal joints, by applying the principle of the SAM method and assuming that genes that function during the effector phase would be similarly activated in both models despite the differences in etiology. The fold change in expression for each gene and the significance levels (*q *values) were estimated based on SAM, assuming that the gene expression profiles were identical between two models. A large population of 594 spots was significantly enhanced, and four spots were suppressed, indicating that the expression levels of these genes changed in both models. Because a number of the spots on the array contained oligonucleotides derived from different clones of the same gene, these spots were found to include a total of 554 non-redundant genes (Additional File 2). The log-transformed (base 2) normalized expression intensities of HTLV-I-Tg mice were then plotted against those of IL-1Ra-KO mice (Figure 1b). The gene expression levels of HTLV-I-Tg mice and IL-1Ra-KO mice were quite similar with a high correlation coefficient (*r *= 0.91), supporting the assumption that a panel of specific genes was similarly activated in both RA models and successfully extracted common, differentially expressed genes.

### Genes overexpressed in the synovial tissues of RA model mice

The expression of *Saa3 *increased maximally (approximately 62-fold) in this study. *Saa1 *and *Saa2*, members of the same family, were also significantly upregulated. Many chemokine genes were also activated, including *Cxcl5 *(LIX/human ENA-78), *Cxcl1 *(KC/human Gro-α), *Cxcl13 *(BLC), *Ccl8 *(MCP-2), *Ccl7 *(MCP-3), *Ccl2 *(MCP-1), *Cxcl14 *(MIP-2γ)*, Ccl5 *(RANTES), *Cxcl16 *(SR-PSOX), *Ccl9 *(Mip-1γ), *Ccl6 *(C10), and *Cxcl12 *(SDF-1). Genes encoding chemokine receptors, such as *Ccr5*, *Ccr6*, *Ccr1*, *Ccr2*, *Cxcr2 *(IL-8Rβ), and *Cxcr4*, were also enhanced significantly. The proinflammatory cytokine *Il1b *(IL-1β) and its receptor *Il1r1 *(CD121a) were upregulated. Although the expression of multiple cytokine receptors, including *Tnfrsf1b *(TNF-αR/CD120b),*Il6ra *(CD126), *Il17r *(CDw217), *Il4ra *(CD124), *Ifnar2 *(IFN-α/βR), *Csf2rb1 *(commonβ/CDw131), *Csf2rb2 *(granulocyte-macrophage colony-stimulating factor [GM-CSF]/IL-3R), and *Csf3r *(G-CSFR/CD114), increased, expression of their ligands could not be detected by our analysis. TNF and TNF-R family genes, such as *Tnfsf3 *(LTβ), *Tnfsf11 *(RANKL/ODF), *Tnfsf31 *(Pglyrp), *Tnfrsf5 *(CD40), and *Tnfrsf21 *(DR6), were significantly elevated. The expression of genes encoding growth factor and growth factor-related proteins, including *Mdk*, *Il18bp*, *Grn*, *Tnfaip6*, *C1qtnf6*, *Fgf10*, *Igf1*, *Igfbp4*, *Igfbp7*, *Pdgfrl*, and *Bmp1*, were also enhanced. In addition to these chemokine/chemokine receptor and cytokine/cytokine receptor genes, several genes were identified that were upregulated more than 10-fold in comparison with WT mice. These genes include *Igh-4 *(Serum IgG1) and *Mmp3 *(Stromelysin-1), which are transcripts known to be increased in RA [23,24]. Although the expression of *Glipr2*, *Kcnj15*, and *Col4a2 *were also elevated, no augmentation of these genes has been previously reported in patients with RA. Moreover, major histocompatibility complex (MHC) class I genes (*H2-D1*,*H2-Q8*, and *H2-Q10*) and MHC class II genes (*H2-DMa*, *H2-DMb1*, *H2-Aa*, *H2-Ab1*, *H2-Ea*, and *H2-Eb1*) were also significantly detected in this data set. Using northern blot hybridization techniques, we examined the expression of some of these genes, including key cytokines and cytokine receptors (*Il1b*,*Il1r1*,*Il-6ra*), chemokines and their receptors (*Cxcl5*,*Cxcl1*,*Cxcr4*), class I and class II MHC genes, and several selected genes, and confirmed the augmented expression of these genes ([3,4] and unpublished data about novel genes).

### Density of the arthritis-related genes within each chromosome

**Figure 2:**
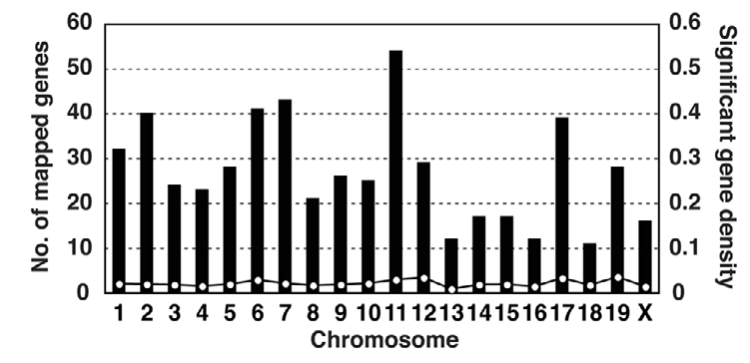
Distribution of arthritis-related genes on the mouse genome. The numbers of significantly changed genes are indicated for each chromosome (closed bars). The density of significantly changed genes (Number of significantly changed genes/Total number of genes on the chromosome estimated from the data of Mouse Genome Search in The Bioinformatics Analytical Toolkit; see Materials and methods) is shown for each chromosome (open circles).

Many functionally related genes form clusters on chromosomes [25]. Because these functionally related genes might be activated simultaneously upon induction, we analyzed the gene expression changes of gene clusters by using the microarray data. Using the public genome database, we located 538 of the 554 significant genes at the specific chromosomal regions. The numbers of the significant genes were plotted against each chromosome (Figure 2), giving a maximal number for chromosome 11, which included 54 genes. A second peak in gene number was found on chromosome 7, which included 43 genes. The third peak on chromosome 6 included 41 genes. Subsequently, chromosomes 1, 2, and 17 were determined to have many significant genes. The density of the significant genes was similar among all chromosomes, with the gene density of 0.022 ± 0.007 (Number of significantly changed genes/Total number of the genes in the chromosome) (shown by open circles in Figure 2). Thus, genes significantly upregulated in arthritis were broadly distributed throughout the genome.

### Transcriptome mapping of arthritis-related genes

**Figure 3:**
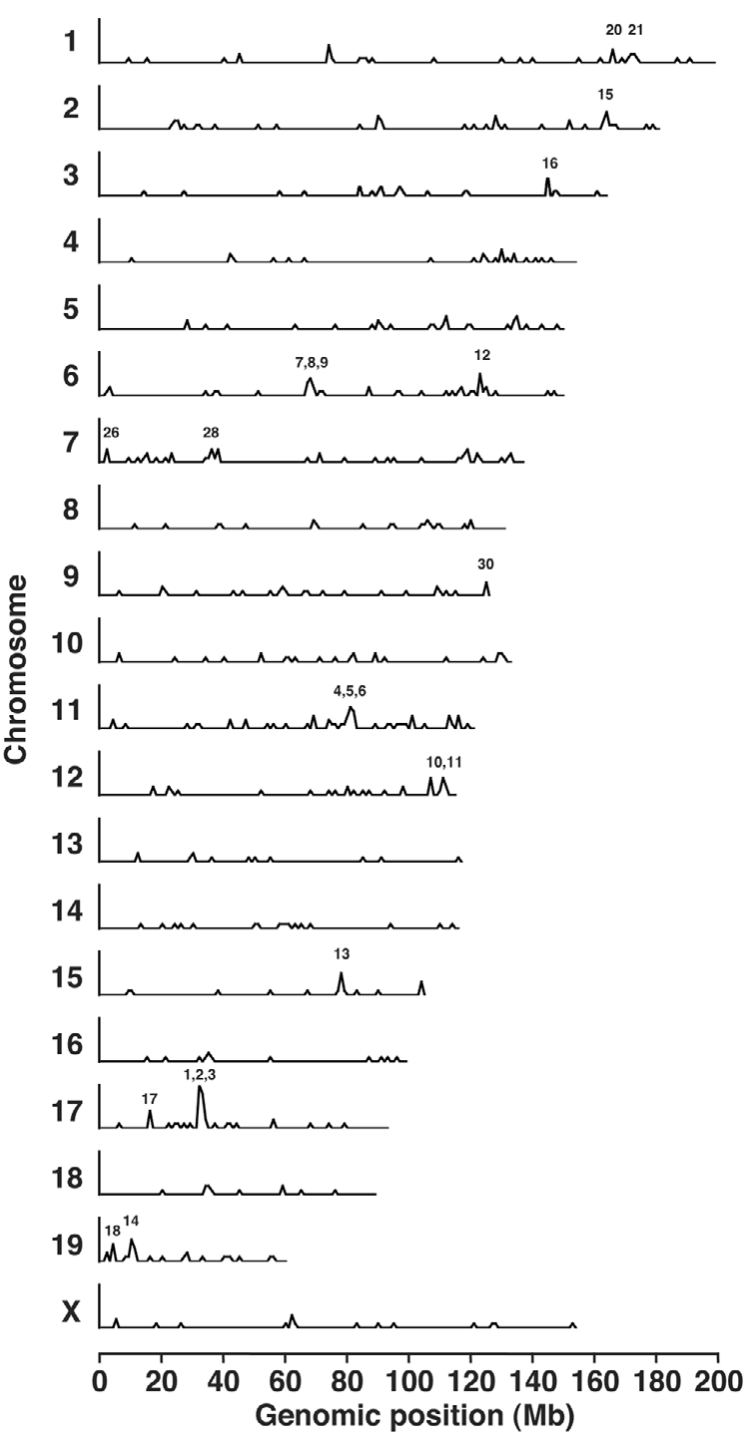
Transcriptome mapping of arthritis-related genes. Significantly changed genes were mapped in every 1-megabase (Mb) interval on each chromosome. The chromosome number is indicated in each panel; the peak number corresponds to the number given in Table 1.

**Figure 4:**
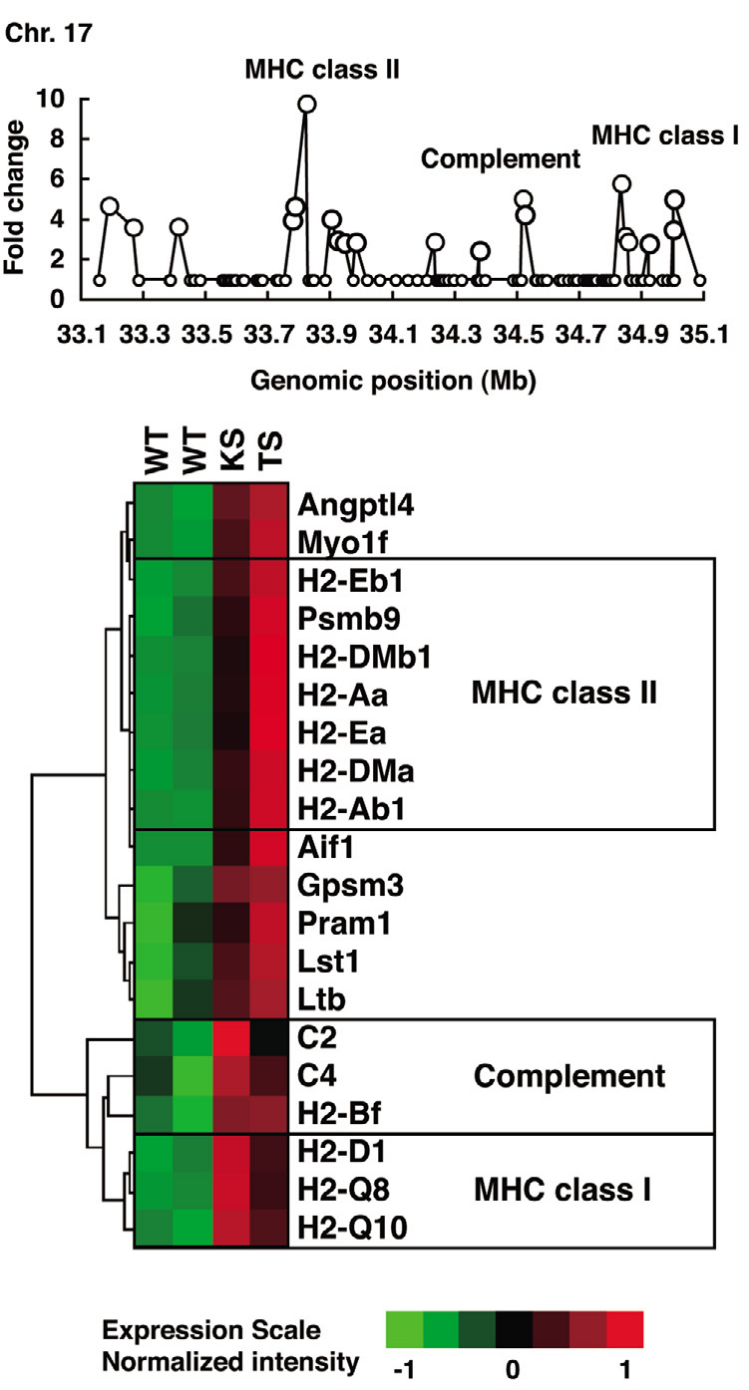
Identification of the *H-2 *gene cluster as one of the significantly activated gene clusters. The maximal gene density was mapped to the chromosome (Chr.) 17, 33- to 35-megabase (Mb) locus (#1–3), which corresponds to the *H-2 *gene cluster. Fold changes of the significantly changed genes are shown in a region of 2-Mb window. Hierarchical clustering of these genes is visualized below. Each column represents an RNA preparation from a different mouse strain, and each row represents an individual gene. Red represents expression levels greater than the median, and green represents those less than the median. The expression scale is shown at the bottom. KS, interleukin-1 receptor antagonist-knockout mice; MHC, major histocompatibility complex; TS, human T-cell leukemia virus type I- transgenic mice; WT, wild-type mice.

**Figure 5:**
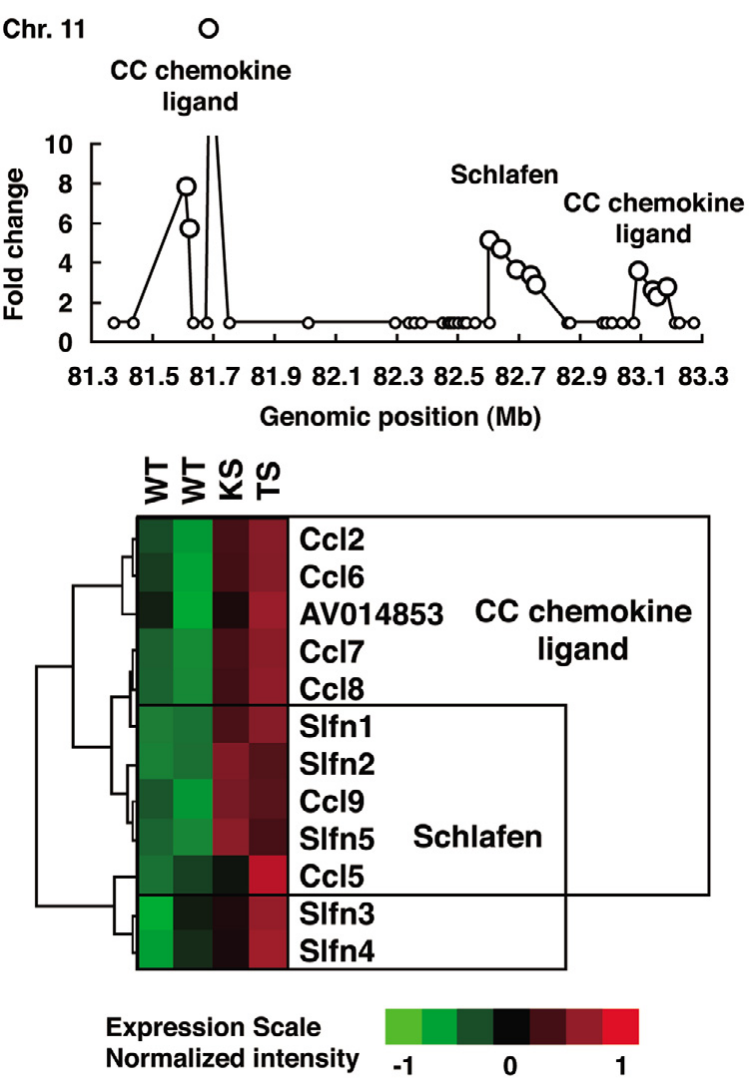
Arthritis-related gene clusters on chromosome (Chr.) 11. The peak at the Chr. 11, 81- to 83-megabase (Mb) (#4–6) includes *Ccl *and *Slfn *family clusters. An expanded view of this region in 2-Mb window is shown. Hierarchical clustering patterns of the expression levels are shown below. *Ccl *and *Slfn *genes are clearly clustered in specific narrow loci and augmented in arthritis. CC, Cysteine-Cysteine type; KS, interleukin-1 receptor antagonist-knockout mice; TS, human T-cell leukemia virus type I- transgenic mice; WT, wild-type mice.

**Figure 6:**
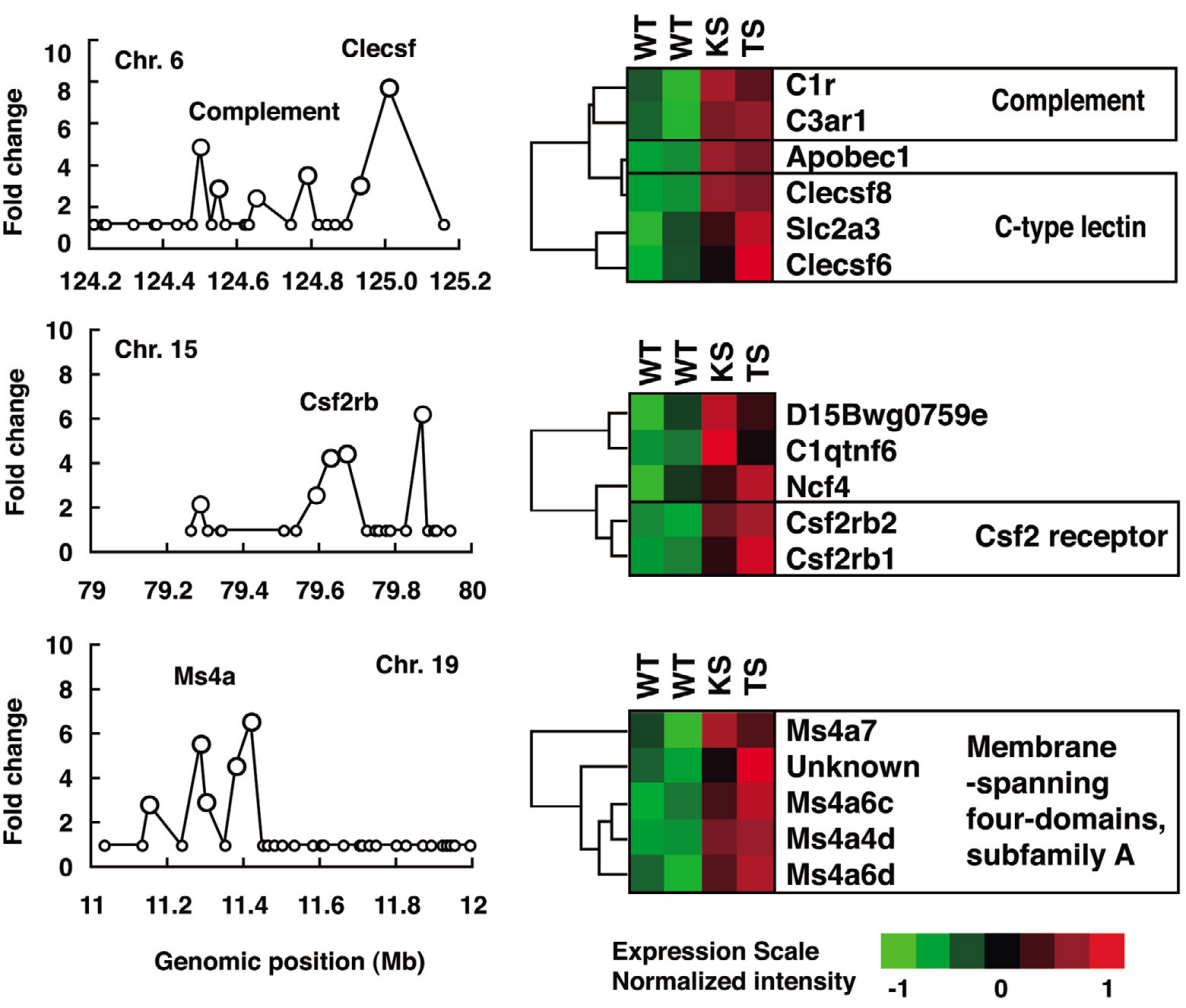
Arthritis-related gene clusters on chromosomes (Chr.) 6, 15, and 19. The peaks on the Chr. 6, 124-megabase (Mb) locus (#12) includes complement receptor genes and C-type lectin superfamily (*Clecsf*) genes, the Chr. 15, 79-Mb locus (#13) includes colony-stimulating factor 2 receptor beta (*Csf2rb*) genes, and the Chr. 19, 11-Mb locus (#14) includes membrane spanning four-domain, subfamily A (*Ms4a*) genes. Expanded views of these loci in 1-Mb scale and hierarchical clustering of the expression levels of these genes are shown.

To identify individual gene clusters, significantly changed genes were mapped into the whole genome in 1-Mb scale windows. Figure 3 shows the distribution of the arthritis-related genes across the entire mouse genome; the gene clusters derived from this mapping are shown in Table 1. For convenience, peaks were numbered, and the numbers in parentheses below indicate the peaks in Figure 3. The map allocates many of the clusters of highly expressed genes to specific chromosomal regions. The maximal gene density was mapped to the chromosome 17, 33-Mb locus (#1) and neighboring 34-Mb (#2) and 35-Mb (#3) regions, which corresponds to the *H-2 *gene cluster. An expanded view of this region, shown in Figure 4, included 20 genes in a 2-Mb scale. The *H-2 *gene cluster includes a number of MHC class I, MHC class II, and complement genes. Hierarchical clustering and visualization of these genes classified these significant genes into three major functionally related gene clusters, including MHC class II cluster (*H2-Dmb1*, *H2-Aa*, *H2-Ea*, *H2-DMa*, *H2-Ab1*, and *H2-Eb1*), complement cluster (*C2*, *C4*,*H2-Bf*) and MHC class I cluster (*H2-D1*,*H2-Q8*,*H2-Q10*). The expression of additional immune-related genes, including *Psmb9 *(proteasome) and *Ltb *(lymphotoxin β), was also increased significantly. Another peak was on chromosome 11, 82-Mb locus (#5) and neighboring 81-Mb (#4) and 83-Mb (#6) locus, which corresponds to the *Slfn *and *Ccl *gene cluster. An expanded view of this region, shown in Figure 5, included 11 genes in a 2-Mb scale. Hierarchical clustering and visualization of these genes, including CC chemokine ligand genes (*Ccl2*, *Ccl6*, *Ccl7*, *Ccl8*, *Ccl9*, and *Ccl5*) and schlafen genes (*Slfn1*, *Slfn2, Slfn5, Slfn3*, and *Slfn4*), are shown. Moreover, chromosome 6, 68- to 70-Mb locus (#7–9) and chromosome 12, 112- and 113-Mb locus (#10,11) formed a cluster of immunoglobulin genes, kappa chain and heavy chain, respectively. High peaks were also detected at chromosome 6, 124-Mb locus (#12), which includes several genes of complement receptor and C-type lectin superfamily, and chromosome 15, 79-Mb locus (#13), which includes colony-stimulating factor 2 receptor beta (*Csf2rb*) genes, and chromosome 19, 11-Mb locus (#14), which includes membrane-spanning four-domain, subfamily A (*Ms4a*) genes. An expanded view of these loci, shown in Figure 6, included five genes in a 1-Mb scale, respectively. Chromosome 6, 124-Mb locus (#12) includes clusters of complement genes (*C1r*, *C3ar1*) and C-type lectin superfamily members (*Clecsf6*, *Clecsf8*). (*Clecsf8 *mapped into the 125-Mb locus but was next to *Clecsf6*.) Chromosome 15, 79-Mb locus (#13) includes two *Csf2rb *genes (*Csf2rb1 *and *Csf2rb2*). Chromosome 19, 11-Mb locus (#14) corresponds to the *Ms4a *gene cluster (*Ms4a7*,*Ms4a6c*,*Ms4a4d*,*Ms4a6d*, and an unknown gene). Although no gene family is detected in chromosome 2, 165-Mb locus (#15) and chromosome 19, 5-Mb locus (#18), chromosome 3, 146-Mb locus (#16) and chromosome 17, 17-Mb locus (#17) included gene clusters of guanylate nucleotide binding protein and formyl peptide receptor, respectively. Additional clusters of arthritis-related genes were identified, including a cluster of selectin genes on chromosome 1, 167-Mb locus (#20), Fc receptor cluster on chromosome 1, 174- and 175-Mb locus (#21), a paired-Ig-like receptor cluster on chromosome 7, 3-Mb locus (#26), serum amyloid A cluster on chromosome 7, 39-Mb locus, and the gene cluster of CC chemokine receptor on chromosome 9, 126-Mb region (#30).

## Discussion

To identify genes involved in the pathogenesis of arthritis, we used two mouse models of RA. We compared the gene expression profiles between arthritic and normal joints using these models and high-density oligonucleotide arrays, in which approximately 36,000 genes and ESTs were analyzed. We analyzed whole synovial tissues instead of using specific cell types because we are interested in not only expression level changes of a gene in a cell but also cell population changes in the synovial tissues [26]. In this report, we examined two mouse models of RA on the same genetic background and with the same disease severity. These models, however, had different etiologies; one is caused by the action of HTLV-I-*tax*, whereas the other is caused by excessive IL-1 signaling. We extracted common genes that were involved in the pathogenesis of both models irrespective of etiology. We expected to identify genes involved in the effector phase of the disease, given that the molecular mechanisms of the initial phase are likely to be different between the two models. With RNA samples derived from the arthritic joints of either animal model, 1,467 clones on the array (approximately 4%) changed at least threefold. The SAM method demonstrated that, of these genes, 554 independent genes changed significantly in both models. These results suggest that a large proportion of the genes functioned in the pathogenesis of arthritis in both models. These common genes may function during the effector phase, whereas those specific to the individual models may function during the initiation phase in a manner dependent on the disease etiology. We analyzed those genes whose changes in expression levels were common to both models.

We found that several interesting genes, including *Saa3*, which encodes serum amyloid A3, were activated in these models. This gene was upregulated to the greatest extent in arthritic mice in comparison with normal mice. IL-1β induces *Saa3 *expression; SAA3, in conjunction with SAA1/SAA2, the expressions of which were also elevated in our RA animal models, induce the transcription of matrix metalloproteinases (MMPs) [27]. *Mmp-3 *and *Mmp-9*, two such MMPs, were also upregulated in these models. We also observed the upregulation of chemokines, such as *Cxcl5 *and *Cxcl1*, which recruit neutrophils to inflammatory sites through interactions with their receptor, *Cxcr2*, which was also elevated and confirmed by northern blot analysis. *Cxcr4 *may be involved in the chemotaxis of naïve and memory T cells. *Ccr1 *and *Ccr2 *are expressed on memory T cells, whereas *Ccr5 *is expressed on Th1 cells. *Cxcl12*, the ligand for *Cxcr4*, is produced in the synovial tissues of patients with RA [28]. *Ccr6*, *Ccr1*,*Ccr2*, and *Ccr5 *are specifically expressed by immature dendritic cells (DCs). *Cxcl16*, a T-cell chemoattractant, is expressed by both DCs and macrophages. *Ccl9*, *Ccl6*, and *Cxcl13 *are produced by macrophages, whereas *Cxcl14 *is produced by fibroblasts. In addition, proinflammatory cytokines and their cognate receptors, such as IL-1^β^, IL-1RI, TNF-α R, IL-6Rα, IL-2R^γ^, and IL-17R, were also significantly elevated. Thus, the augmented expression of chemokines, cytokines, and their receptors indicates the importance of these molecules in the pathogenesis of arthritis. It should be noted, however, that the augmentation of the expression of these genes does not necessarily mean that they are actually activated in synovial tissues. The infiltrated cells to the inflammatory sites may also contribute to the expression pattern. Elevated expression of serum amyloid proteins, metalloproteinases, chemokines, and cytokines is frequently seen in the joints of patients with RA [27,29], indicating that the gene expression profiles obtained from these RA models well represent those of patients with RA. In addition, many of these genes function during the elicitation of inflammation, supporting our assumption that the genes augmented in both models may function during the effector phase.

The gene expression profiles of the well-known collagen-induced arthritis (CIA) model [30] and proteoglycan-induced arthritis (PGIA) model [31] for RA have already been reported. We compared our data set with those previously reported gene expression profiles. Approximately 60% of the genes that changed in our models also changed more than two times at the early phase of CIA. Moreover, approximately 63% of the genes corresponded to those found in PGIA at the acute phase and approximately 50% at the initiation and chronic phases. Thus, many of the genes that changed in our models also changed in other RA models, suggesting similar mechanisms function in common in those RA models.

We next mapped these arthritis-related genes into chromosomes. Working under the assumption that functionally related genes form clusters, we attempted to detect the activation of genes as a cluster, despite the fact that small individual changes were not prominent enough to be detected in our initial analysis. The most significant peak was detected at chromosome 17, 33- to 35-Mb locus (#1–3), which corresponds to the *H-2 *gene cluster. Other significant peaks corresponded to chromosome 11, 81- to 83-Mb locus (#4–6), which includes members of the *Ccl *and *Slfn *families. Immunoglobulin kappa chain cluster in chromosome 6, 68- to 70-Mb locus, and heavy chain cluster in chromosome 12, 112- and 113-Mb locus were also clearly detected. Moreover, chromosome 6, 124-Mb locus, chromosome 15, 79-Mb locus, and chromosome 19, 11-Mb locus were included in those attractive gene clusters. The contribution of individual genes in these clusters was relatively small; the significance of a number of genes was recognized only after summation of genes in a specific region. These gene clusters, however, clearly contained a number of genes important in the development of arthritis. For more than two decades, the MHC gene cluster has been known to affect susceptibility to a variety of autoimmune diseases [32,33]. This region encodes the MHC class I and class II genes and other immune-related genes. MHC molecules are required for antigen recognition by lymphocytes, ultimately leading to activation and progression of immune responses. In addition, the genes encoding complement components are located within this cluster. The levels of antibodies against IgG and type II collagen were elevated in these RA models [4,7]. In these animal models, the classical pathway components of the complement system, C2, C4, encoded within this cluster (Chr. 17, 34 Mb: [#2]), and C1q, C1s, and C1r, found within other clusters, were upregulated, suggesting that immune complexes are involved in this enhancement of gene expression. The expression of Factor B, encoded by the *H2-Bf *gene within this cluster, was also augmented. Factor B is an essential component of the alternative pathway of the complement system. This pathway is also critical in K/BxN mice, another animal model for RA [34]. Although the alternative pathway typically activated by microbial surface antigens, immune complexes formed by autoreactive IgGs initiate the alternative pathway in these models to facilitate the development of arthritis.

A similar study was reported using CIA and PGIA models, identifying gene clusters in chromosomes 2, 3, 11, and 17 [35]. Their microarray chips contained a total of 9,500 known genes and EST clones, and 203 selected genes were mapped into the chromosome using a 1.5-fold differential expression threshold level. In the present study, 36,000 oligonucleotide probe sets were included in microarray chips, and 550 selected common significant genes were mapped. Interestingly, we detected the same clusters in chromosomes 2, 11, and 17 as were found in their study, suggesting that the same genes are involved in the pathogenesis of arthritis regardless of the etiology. Although they found a cluster on chromosome 3 containing 11 genes within an 8-Mb-long region, this region was not assigned as an arthritis-related gene cluster in our study, because of broad distribution of genes among cytobands. Increased expressions of MHC class I and class II genes, complement genes, and chemokine genes were already reported using microarray analysis ofsynovium from patients with RA [36] or streptococcal cell wall-induced arthritis in rats [37]. Our results in this report using mouse arthritis models are consistent with these results, suggesting that these genes are commonly involved in the pathogenesis at the elicitation phase.

The gene density peak on chromosome 11, 81- to 83-Mb locus (#4–6) includes the *Ccl *and *Slfn *genes. CCL2, CCL7, and CCL8, members of the chemokine family, recruit monocytes to sites of injury and infection. CCL2 influences innate immunity through this effect on monocytes and modulates adaptive immunity via control of Th2 polarization. An antagonist of CCL2 suppresses arthritis in the MRL-*lpr *mouse model [38]. CCL5, a T-cell and monocyte chemoattractant, plays an important role in the development of adjuvant-induced arthritis [39]. CCL6 was expressed in experimental inflammatory demyelinating disorders that promote recruitment of macrophages [40]. CCL9 recruits CD11b^+ ^DCs and promotes osteoclast differentiation and survival [41,42]. Thus, these chemokines likely play important roles in the development of arthritis. Schlafen proteins have been implicated in the regulation of cell growth and T-cell development [43]. These proteins form a big family localized to a specific genomic cluster adjacent to the *Ccl *gene cluster. This region coincides with a number of autoimmune susceptibility loci, including the *Idd4*, *Eae7*, and *Orch3 *loci [44]. The syntenic region of this locus on 17q of the human genome is associated with several autoimmune disorders [45]. Therefore, this genomic region on chromosome 11 is likely to be critical in the development of autoimmune arthritis.

The chromosome 6, 124-Mb (#12) locus is one of the non-MHC susceptibility loci linked to complex autoimmune diseases. The mouse 6 F2 cytoband, the syntenic rat 4q42 and human 12p13 regions, have been implicated in several inflammatory diseases, including arthritis [46-48], systemic lupus erythematosus, spontaneous diabetes, atheroscrelosis, encephalomyelitis, asthma or airway hyper-responsiveness, and allergy. This locus contains complement component genes and C-type lectin superfamily genes, suggesting the possible involvement of these protein products in the development of arthritis. Chromosome 15, 79-Mb locus (#13) contains GM-CSF receptor, *Csf2rb1*, and *Csf2rb2*. It is known that GM-CSF plays an important role in the effector phase of arthritis [49]. *Ms4a *family molecules, CD20 (*Ms4a1*), and high-affinity IgE receptor ^β ^chain (FcΣ RIb; *Ms4a2*) tended to be upregulated in these animals, but these genes were not significantly altered in this study. Although the upregulation of *Ms4a7*, *Ms4a4d*, *Ms4a6d*, and *Ms4a6c *expression in chromosome 19, 11-Mb locus (#14) was also observed in these models of RA, the roles of these molecules in the development of arthritis are not yet known [50].

We succeeded in identifying several arthritis-related genes by transcriptome mapping, but we might have failed to detect some potentially important genes. Because we analyzed mRNA preparations from whole joints, but not from a single species of cell, we could not detect the change of gene expression in a single species of cell when the content of this single species of cell was low in the whole joint. Furthermore, we could not detect the MMP gene cluster consisting of MMP-1a, MMP-1b, MMP-3, and MMP-13 on chromosome 9, which were suggested to be involved in the pathogenesis [51]. In this case, we demonstrated upregulation of MMP-3 and MMP-9 in both HTLV-I-Tg and IL-1Ra-KO mice. However, MMP-8 and MMP-13 expressions were increased more than three times in IL-1Ra-KO mice, but not in HTLV-I-Tg mice, excluding the *MMP-8 *and *MMP-13 *genes from significantly activated genes in this analysis. Unfortunately, MMP-1a and MMP-1b were not mounted on the GeneChip we used (Murine Genome U74v2 Set). Nonetheless, the present data are important for understanding the transcriptome change as a whole, reflecting the gene expression levels not only in a single cell but also in the cell population in the joints during arthritis.

## Conclusion

We have performed a comprehensive transcriptome analysis of two etiologically different RA models by using microarrays and identified many genes that were commonly activated in both models. Because the initial mechanism of the pathogenesis should be different between two models, these genes are likely to function during the effector phase of the disease. Moreover, by mapping genes with marginal expression changes into chromosomes, we succeeded in detecting several gene clusters that may be involved in the pathogenesis of arthritis. A similar approach, using microarrays, will likely be useful in detecting genes related to multiple disease processes.

## Abbreviations

CIA = collagen-induced arthritis; Csf2rb = colony-stimulating factor 2 receptor beta; DC = dendritic cell; EST = expressed sequence tag; GM-CSF = granulocyte-macrophage colony-stimulating factor; HTLV-I = human T-cell leukemia virus type I; Ig = immunoglobulin; IL-1Ra = interleukin-1 receptor antagonist; KO = knockout; KS = knockout severe; MHC = major histocompatibility complex; MMP = matrix metalloproteinase; Ms4a = membrane-spanning four-domains, subfamily A; PGIA = proteoglycan-induced arthritis; RA = rheumatoid arthritis; SAM = significance analysis of microarrays; SD = standard deviation; Tg = transgenic; TS = transgenic severe.

## Competing interests

The authors declare that they have no competing interests.

## Authors' contributions

NF, SS, and YI participated in the design of the study. SS prepared the RNA samples and carried out northern blot hybridization with NF. NF carried out the analysis of the microarray data, developed the transcriptome mapping analysis method, and drafted this manuscript as a part of his doctoral thesis. YI planned, designed, and organized the study and finalized the manuscript. All authors read and approved the final manuscript.

## Supplementary Material

Additional File 1 An Excel file containing a table that lists the first 1,467 selected genes, the expression of which changed more than three times in one of the mouse models. Click here for file

Additional File 2 An Excel file containing a table that lists five hundred fifty-four non-redundant genes, the expression of which significantly changed in both models. Click here for file

## Figures and Tables

**Table 1:** Identified arthritis-related gene clusters by transcriptome mapping

**ID**	**Chr.**	**Position (Mb)**	***n *of genes**	* *n *of total genes	**Included gene families**
1	17	33	10	48	MHC class II
2	17	34	8	67	Complement
3	17	35	2	40	MHC class I

	17	33–35	20	155	MHC class II/Complement/MHC class I

					
4	11	81	3	10	CC chemokine ligand
5	11	82	5	25	Schlafen
6	11	83	3	23	CC chemokine ligand

	11	81–83	11	58	Schlafen/CC chemokine ligand

					
7	6	68	3	19	Immunoglobulin kappa chain
8	6	69	4	16	Immunoglobulin kappa chain
9	6	70	2	13	Immunoglobulin kappa chain

	6	68–70	9	48	Immunoglobulin kappa chain

					
10	12	112	4	8	Immunoglobulin heavy chain
11	12	113	2	3	Immunoglobulin heavy chain

	12	112–113	6	11	Immunoglobulin heavy chain

					
12	6	124	5	23	Complement/C-type lectin superfamily
13	15	79	5	27	Colony stimulating factor 2 receptor, beta
14	19	11	5	32	Membrane spanning four-domain, subfamily A
					
15	2	165	4	32	-
16	3	146	4	10	Guanylate nucleotide binding protein
17	17	17	4	11	Formyl peptide receptor
18	19	5	4	45	-
					
19	1	75	3	15	-
20	1	167	3	16	Selectin
21	1	174	3	20	(Fc receptor)
22	2	91	3	26	-
23	2	129	3	18	-
24	4	131	3	18	-
25	5	136	3	38	-
26	7	3	3	28	Paired-Ig-like receptor
27	7	37	3	46	-
28	7	39	3	29	Serum amyloid A
29	7	120	3	30	-
30	9	126	3	15	CC chemokine receptor
31	11	70	3	30	-
32	11	102	3	37	-
33	11	114	3	33	-
34	11	117	3	23	-
35	15	105	3	25	-
36	19	12	3	33	-
37	X	63	3	40	-

*Numbers of total genes in the corresponding 1 megabase were obtained from a Mouse Genome Search in The Bioinformatics Analytical Toolkit [20]. CC, Cysteine-Cysteine type; Chr., chromosome; Fc, Fragment crystallizable; ID, identification number; Ig, immunoglobulin; Mb, megabase; MHC, major histocompatibility complex.
